# Optimizing blood biomarkers collection for Alzheimer’s disease among older adults in rural Kenya

**DOI:** 10.1002/alz.091385

**Published:** 2025-01-09

**Authors:** David Ndetei, Pascalyne Nyamai, Christine W. Musyimi, Diana Kasomo, Victoria Mutiso

**Affiliations:** ^1^ Africa Mental Health Research and Training Foundation, Nairobi Kenya; ^2^ University of Nairobi, Nairobi Kenya

## Abstract

**Background:**

Plasma/serum measurements, which stand as the clinical gold standard biomarkers for Alzheimer’s disease (AD) are minimally invasive, facilitating easy collection and processing particularly in low‐resource settings. Often, both laboratory and non‐laboratory medical personnel find themselves collecting these blood biomarker samples in remote hospitals characterized by a high influx of patients, leaving them with limited time and resources (e.g. freezers) for immediate sample processing, transport, and storage. This abstract describes blood biomarker sample collection and storage for AD research among older adults in Makueni County, Kenya.

**Methods:**

In optimizing blood biomarkers collection for AD research among hard‐to‐reach older adults, a multidisciplinary team is crucial. Blood was collected into three Ethylenediamine tetraacetic acid (EDTA) of 10mls each. To ensure the preservation of biomarker integrity, we focused on refining sample handling processes, and maintained the required temperature for sample transportation.

**Results:**

Following sample collection, one of the three EDTA blood tubes was immediately stored in dry ice (‐80 degrees) for Deoxyribonucleic acid (DNA) extraction. A volume of 0.5ml whole blood was taken from one of the remaining two EDTA tubes. The rest was centrifuged at 3400 rpm for 10 minutes and plasma aliquots obtained and put into the barcoded storage vials prelabeled. To preserve the integrity of the blood biomarkers, the collection and transportation process necessitated the use of dry ice (‐80 degrees) with continuous monitoring. This not only ensured the retention of blood biomarkers but also the importance of stability in these biomarkers. Subsequently, during storage, temperature monitoring becomes imperative. The options ranged from manual temperature monitors, albeit with inherent limitations, to more reliable yet costly data loggers. This monitoring was crucial to guarantee the integrity of the stored samples and maintain the accuracy of biomarker analysis.

**Conclusion:**

The transportation of blood samples from the rural hospital to centralized facilities with adequate freezers demands meticulous cold chain maintenance. Clear guidelines encompassing sample collection, transport, processing, and storage, in hard‐to‐reach older adults should be delineated for future biomarker research in AD research.

